# Experiences of stigma and discrimination endured by people suffering from schizophrenia

**DOI:** 10.4103/0019-5545.39758

**Published:** 2008

**Authors:** Santosh Loganathan, Srinivasa R Murthy

**Affiliations:** Department of Psychiatry, National Institute of Mental Health and Neurosciences (NIMHANS), Bangalore, India

**Keywords:** Experiences, schizophrenia, stigma and discrimination

## Abstract

**Objective:**

It is important to understand stigma in India, given its varied culture and mixture of rural and urban populations. Information from western literature cannot be applied without considering the sociocultural differences.

**Aims:**

The research aimed to study the subjective experiences of stigma and discrimination undergone by people suffering from schizophrenia in rural and urban environments in India.

**Settings and Design:**

Patients were selected from the outpatient services of six adult psychiatric units of the National Institute of Mental Health and Neurosciences (NIMHANS), India, and from the six outreach centers located in rural areas.

**Materials and Method:**

Two hundred patients diagnosed with schizophrenia were selected from rural and urban areas. The experiences of stigma and discrimination were assessed using a semi-structured instrument.

**Statistical Techniques:**

Both quantitative and qualitative analyses were done.

**Results:**

Significant differences were seen between rural and urban respondents. Urban respondents felt the need to hide their illness and avoided illness histories in job applications, whereas rural respondents experienced more ridicule, shame, and discrimination. The narratives provide direct views of patients, supporting the key findings.

**Conclusion:**

Mental health programs and policies need to be sensitive to the consumers’ needs and to organize services and to effectively decrease stigma and discrimination.

## INTRODUCTION

More than 40% of countries have no mental health policy, and over 30% have no mental health programs. Existing health plans frequently do not cover mental and behavioral disorders at the same level as other illnesses, creating significant economic difficulties for patients and their families. One of the identified reasons for low support for mental health is the stigma attached to mentally ill individuals.[1]

In the past decade, several professional associations have initiated awareness campaigns on mental illness. In devoting The World Health Day 2001 and the World Health Report 2001 to mental health, the World Health Organization (WHO) stated that mental illness was ignored and mental health is essential to the overall well-being of individuals, societies, and countries.[1] The American Psychiatric Association Assembly and the Board of Trustees[2] approved a Position Statement on discrimination against persons with previous psychiatric treatment to facilitate their full participation in society. In the United Kingdom, the Royal College of Psychiatrists and the Royal College of General Practitioners launched a Defeat Depression Campaign in 1992, which aimed to increase public and professional awareness of depression and its treatment. “Changing minds” organized by the Royal College of Psychiatrists in the United Kingdom[3] imparted information to the public so as to dispel myths and stereotypes about those with mental illness. In 1996, the World Psychiatric Association (WPA) launched the “Open the Doors” initiative.[4] The goal of this program was to increase awareness and knowledge of the nature of schizophrenia and treatment options; to improve public attitudes towards those who have or have had schizophrenia and their families and to generate action to eliminate discrimination and prejudice. The program explored the experiences that patients and their families had gone through. This served to choose goals for interventions to reduce stigma and discrimination.

### REVIEW OF LITERATURE

#### International research findings

Research on stigma and mental illness is gaining importance, and the literature available is exhaustive. However, only studies relevant to themes involved in this research have been reviewed here. Findings from international research (in the past two decades) on stigma have been summarized in Table 1.

**Table 1:** Selected international research findings

Thematic content	Author, year	Findings	Implications
Public services	Watson *et al*., 2004	Police view mentally ill as less responsible,more deserving of pity, more worthy of help, Increased perception of violence[5]	Though study did not replicate real life situation, hostile approach can aggravate circumstances
Educational institutes	Corrigan *et al*., 2005	In contrast to adults, adolescents reported that contact led to more discrimination[6]	Possibly of adolescents having less pre existing information, teenagers experience with stereotype
Family	Phillips *et al*., 2002	Close association between high expressed emotion and perceived effect of stigma[7]	Intervention aimed at either problem may relieve both
Media
a) Movies	Schneider, 1987	Serious films outnumbered by exploitation/horror films[8]	False picture of psychiatrists' work has been presented to the public
b) Newspaper	Day *et al*., 1986	Newspaper portray mentally ill in a negative fashion[9]	Portrayal as a group devoid of positive social identity
Work setting	Manning and White, 1995	Most employers cautious in employing currently ill, More likely to believe a sick-note declaring a physical than a mental illness, Many employers were ‘unsure ‘in their replies[10]	Reflects ignorance about mental illness and its effect on work ability Being on a medically approved treatment program may enhance employment chances
Interventions	Corrigan *et al*., 2001, 2002	Education yields some positive benefits, Contact with patients yields the greatest results Protest yielded no improvement[11,12]	Apart from imparting knowledge, promoting contact with mentally ill more effective
			Trying to suppress negative attitude actually maintains that knowledge
Coping strategy	Katsching, 2000	Both, stigma acceptance and stigma avoidance are irritating and energy-consuming processes[13]	Both these forms of coping are associated with distress
Health services	Hotopf, 2000	Nature of psychiatrists' job itself can be stigmatizing, So can the side effects of medications prescribed[14]	Psychiatrists must challenge their own prejudices, Can play a role in maintaining and reducing stigma

### BACKGROUND OF THE STUDY

It is necessary to compare the characteristics and extent of stigma in different social and cultural regions. In India, with its cultural diversity and mix of rural and urban environment, there is a need to understand the stigma and discrimination experiences of people with mental illness. Indian studies on stigma have been summarized in Table 2. These studies have examined attitudes of the general public toward mental disorders. A caregiver or family member was usually interviewed for information.[19] Thara *et al.* have done a qualitative study of women with schizophrenia who had broken marriages.[21] This research attempts to address the lacuna of published reports of the subjective experiences of stigma and its consequences, undergone by patients in rural and urban environments in India.

**Table 2:** Summary of Indian studies

Author, year	Result Summary	Implications
Neki, 1966	Public fears and rejects mentally ill; Rural population more tolerant[15]	Absolute need to educate public about mental illness. Empower rural population with adequate knowledge.
Dube, 1970	Misconception, superstition and ignorance; Viewing mental illness as visitation of the evil spirits [16]	Dispelling myths and providing the right information to people
Verghese and Baig, 1974	Majority had positive attitudes; Two-thirds against marital alliance to a family, with a history of mental illness. (Bias in sample: predominantly urban)[17]	Though results were biased due to the sampling, still the need to provide information about marriage and mental illness
Wig *et al*., 1980	Pessimistic community attitudes; Preferred modern health services and later returning to traditional healers[18]	Awareness and education of the public alone can improve negativistic attitudes.
		Traditional healers too need to be aware of facts about mental illness
Thara and Srinivasan, 2000	Marriage, dread of rejection from neighbors, concealing the illness, being a female and younger age of caregiver and patient were most stigmatizing;[19]	Need to dispel myths about marriage and its relation to illness
		Hiding illness from others can have positive and negative implications
Srinivasan and Thara, 2001	Families believing in the supernatural causation of illness less likely[20]	Changing views about causation of mental illness with time, when compared to Dube's study (1970)
Thara *et al*., 2003	Women separated from husbands- but not legally, concerned about their future, being a burden to ageing parents[21]	Stigma attached to separation was as distressing as that of being mentally ill
Murthy *et al*., 2005	Awareness more among literates and urban; Females felt sexual harassment as a consequence of stigma; In general, stigma was due to emphasis on heredity as a cause, fear of violence and need for lifelong care[22]	Need to enhance awareness among rural and illiterate; consolidate gains among the urban.
		Highlights the particular needs of women with mental illness

## MATERIALS AND METHODS

People from rural and urban areas in the age group of 16 to 59 years with a diagnosis of schizophrenia (any subtype) according to ICD (International Classification of Disease) -10 DCR (diagnostic criteria for research) were included in the study. Those with a comorbid Axis II or I disorder, coexisting medical disorder; and symptomatic and uncooperative patients were excluded. Patients who spoke English and/or Kannada fluently were chosen, as the investigator was familiar with reading and administering the scale in these languages. Only those patients whose duration of illness was less than 6 years (as any chronic illness was considered to be associated with stigma) were selected. Those who were asymptomatic for at least a period of 6 months were chosen, as symptoms could interfere with the assessment. Information from the caregivers and the patients’ case file, along with a clinical mental status examination by the treating psychiatrist, was used for diagnostic confirmation. The purpose of the study was explained to each patient, and written informed consent was obtained individually.

### Characteristics of the sample

Two hundred patients diagnosed with schizophrenia were selected. Of these, 151 (100 from urban areas and 51 from rural areas) were selected from the outpatient services of six adult psychiatric units of the National Institute of Mental Health and Neurosciences (NIMHANS), India. The remaining 49 patients were selected from the six outreach centers located in rural areas, through the community psychiatry services of the institute.

### Tools

The instrument used to assess stigma and discrimination experiences is a semi structured scale developed in an earlier study.[22] This instrument was used in over 1,000 patients in four cities, with caregivers to assess stigma, as a part of the Indian initiative of the WPA Program to reduce the stigma and discrimination because of schizophrenia. A factor analysis of this scale was done and the final instrument was arrived at. It consists of two parts: the first part elicits the socio-demographic information of the respondents, and the second part of the scale measures the stigma and discrimination experiences (Appendix 1).

### Statistical techniques

Both quantitative and qualitative analyses were done. The quantitative analysis was done using SPSS (Statistical Package for Social Sciences) version 7.5. Spearman's correlation was applied to find the correlation between various variables, the socio-demographic characteristics, and the stigma and discriminatory experiences. The data from urban and rural areas were analyzed for differences using t-test and chi-square tests.

## RESULTS

### Socio-demographic characteristics (summarized in Table 3)

**Table 3:** Results of socio-demographic characteristics

Socio-demographic data	Rural (%)	Urban (%)
Age (years)		
15-29	28	26
30-45	43	40
46-60	29	34
Gender		
Male	60	58
Female	40	42
Income (Rs.)		
Nil	45	49
<1,000	33	18
>1,000	22	33
Family type		
Single	-	3
Joint	40	23
Nuclear	60	74
Education		
Illiterate	30	9
Schooling	50	45
Higher	20	46
Marital status
Married	64	51
Single	28	33
Others	8	16
Employment		
Employed	49	51
Unemployed	21	22
Homemaker	30	37
Illness type		
Paranoid	74	82
Hebephrenic	1	-
Catatonic	2	-
Undifferentiated	8	11
Residual	4	2
Simple	1	-
Schizophrenia NOS	10	5
Illness duration		
6months-2 year	3	7
2-4 years	57	60
4-6 years	40	33

The urban and rural groups did not differ significantly from each other on age and gender. More people (30%) were nonliterate in the rural sample as compared to the urban sample (9%).

The significant findings are summarized in Table 4, and the related narrative experiences are described. In general, greater stigma was experienced during the acute phase of the illness (93%) than during the stabilization phase.

**Table 4:** Summary of results

Vaiables	Residence		Total *n* (%)	*P* value
			
	Rural (n)	Urban (n)		
Impact of illness on general life				
Hide your illness				
Yes	40	60	100 (50)	0.007
No	60	40	100 (50)	
Relationship of stigma experience and phase of illness				
Acute Phase	92	94	186 (93)	0.6
Common stigma and discrimination experiences				
Ridiculing by others				
Yes	29	16	45 (22.5)	0.041
No	71	84	155 (77.5)
Sources of stigma				
Unacceptable behavior	53	48	101 (50.5)	0.766
Lack of correct knowledge	19	26	45 (22.5)	
Not being able to work	15	14	29 (14.5)	
Supernatural causation	3	1	4 (2)	
Consequences of stigma				
Conceal illness in job applications				
Yes	16	34	50 (25)	0.003
No	84	66	150 (75)	
Attitude of friends and relatives				
Hide from relatives				
Yes	38	51	89 (44.5)	0.087
No	62	49	111 (55.5)	

### Urban residents

“My wife and my in-laws used to call me mad and used to scold me when I had the illness at the severest level. Later, they left me. I don't tell anyone that I have a mental illness, fearing that I will not be respected or be looked down upon by him or her.”

“People ask me, ‘Why aren't you working? What is wrong with you? You are either sitting still or wandering about.’ They tell me ‘He's simply wandering about relying on his children's earnings’”

“People at work call me ‘Half,’ ‘Mental.’ I get angry but I don't react much. People don't respect me. They don't assign me responsibility. I pray to the Lord daily that my children shouldn't get this illness. I feel I can't maintain relationships, and people may think otherwise of me; so I find it difficult at my place of work. I think being mentally ill means a lot of suffering, and they suffer more from the label than the illness. At times, I feel I shouldn't have got this illness. To improve public attitudes about mental illness, we should tell them that all are not dangerous; and by giving right facts and correcting myths, they can be educated. The family members and the relatives too should be educated.”

### Rural residents

“My friends tell me I'm crazy. They say I talk things that are meaningless and that I don't behave well.”

“People show discrimination to show that they are superior to ‘us.’ Especially, in rural areas, it is difficult to educate people about the illness. Advertising through posters in the local language may help.”

“The village people cannot be taught or educated regarding this illness. They have to learn on their own.”

### Consequences of stigma

“It is difficult to open one's mind and let others know that you have a mental illness.”

“I feel as though I can't do anything with my life. I can't remember how I used to work earlier. Since 3 months of taking treatment, I wasn't going out to meet people, for the fear of people knowing about my illness. Nobody knows about my illness. I feel, the moment they get to know, they might look upon me unfairly. When people ask me what is wrong with me, I tell them I have insomnia. Compared to other physical disabilities, a person with a mental illness is at a disadvantage; because unless he is mentally stable, he cannot function.”

“I feel shy that I have a mental illness. People tell me not to go anywhere, not to talk to people. So I too don't speak much to them. When people ask what is wrong with me, I don't tell that I am suffering from a mental illness. I tell them that I have a BP (hypertension) problem.”

“People call me’ ‘mad.’ They see me and try to snatch money from me. They don't allow me to take part in any game with them. At home my father resorts to beating me when he's upset with me. He says I'm just acting ill.”

“People throw stones at me. They tease me and call me names. I used to be a tailor. Now, I cannot do that and work as a waiter in a hotel. My friends too throw coconut shells at me.”

“People say, ‘He's ill, don't talk to him.’ They call me mad. They say, ‘if we talk to him, his illness will come to us. Don't drink the water he has drunk. Don't share his food; eat separately.’ They tease me and laugh at my inability to work.”

“Children around my neighborhood call me ‘loose’ and laugh at me, which makes me angry and feel helpless. I could not work in a garment shop because of my inability to lift heavy weights.”

### The experience of stigma and discrimination in the social and work life

“People would avoid me and I too avoid people due to poor rapport with them since my illness began. I feel I would not perform well. I feel I may need help in my job (marketing) and can't deal with it on my own. My younger brother doesn't respect me, doesn't talk or listen to me, nor does he feel concerned about me. I was called a ‘sick man’ at a social gathering and felt very hurt by it.”

“People consider me dirty. They call me Mentalu (of unsound mind). My neighbors scold me. I am given to eat stale food. My own daughter scolds me and doesn't feed me well.”

“I was fine earlier; now I have become ‘weak.’ When people call me names like ‘mad,’ I feel bad about it. People around my house don't talk to me. They laugh upon seeing me. My parents have refused to give a share of their property to me.”

“People look down upon me and use dirty language when they speak to me. They say that I have to go to a forest and perform penance. Why should such an illness come to poor people like us?”

### Experiences related to marital life

“I was sharp before I got this illness. Now I feel I am a bit dull. People say I behave like a Huchha (madman). I have no peace of mind. My wife did not want to live with me any longer and deserted me forever.”

“My husband left me because I am mentally ill. When I was pregnant, my in-laws got an abortion done on me, saying the children will also be mentally ill. I used to work as a salesgirl, and they told me that I was too slow and that I just stood at the counter and that they could not pay me for just standing there.”

### The experiences of stigma and discrimination in relationship with friends and relatives

“I was to marry my cousin; now after my illness, my relatives have decided not to get her married to me and have got her married to someone else. At home also people don't give me importance; whatever I say has no value at all, and I have difficulty in getting money.”

“Though my illness started after my marriage, my husband says that he's been cheated. He doesn't allow me to go to my mother's place. He does not tell anyone that I have a mental illness, fearing that I will not be respected or will be looked down upon by others.”

## DISCUSSION

Stigma and discrimination were most experienced during the acute phase of the illness. Presence of socially unacceptable behavior was the condition most associated with creation of stigma. Both these findings are interrelated as such; unacceptable behavior is seen predominantly in the acute phase of the illness. These findings are consistent with those of Penn *et al.*,[23] who reported that knowledge of acute phase induced greater stigma. Raguram *et al.*[24] also described that positive symptoms, namely, hallucinatory behavior, delusions, and suspicion, were found to be very distressing, in that order, to the family members. Lack of awareness, difficulty in working, and attribution of a supernatural cause were the other variables, apart from socially unacceptable behavior, that were examined for their association with stigma. Socially unacceptable behavior created more stigma than attribution of a supernatural cause. These findings were similar to those of Srinivasan and Thara,[20] who verified that families living with patients suffering from chronic schizophrenia rarely subscribed to the idea of a supernatural cause.

As the stigma experience of ridicule, shame, and discrimination was more in the rural sample, creating awareness about, and disseminating information regarding, the acute phase of the illness in the rural population will be beneficial. The narrative experiences point towards the difficulty, arising out of rigidity, in educating the rural population, given their attitude of resistance to change. It is thus important to understand their explanatory model of illness and then approach them regarding their attitudes and their impact on the illness. People in urban areas were however open to all types of educational strategies to improve the knowledge of the urban public, including those to promote contact with the patients. Corrigan *et al.*[11] found that promoting contact with the mentally ill led to attitudinal change.

Concerns about disclosure and the impact the illness had on the self-esteem of the caregivers and family members figured as distressing stigma-related perceptions.[24] Thara and Srinivasan[19] too found that the need to hide the illness from others was stigmatizing. Narratives from the urban respondents point to concerns regarding disclosure of their illness and their lowered self-esteem. Phillips *et al.*[7] report that patients’ behavior is observed more in the crowded urban community compared to the rural community, which could perhaps explain the need to conceal their illness. Concealment in order to procure a job, get married, own a house, etc., cannot be ignored. The comparison showed differences between the urban and rural samples, with the rural sample experiencing more ridicule, shame, and discrimination as compared to the urban sample. This could possibly be due to the openness brought about by living conditions in the rural environment, where everyone knows the other. It also emphasizes the need to educate the rural people about the symptoms of the illness, the treatment available, and the process of recovery. Efforts need to be made to educate the public about the ill effects of patients being ridiculed and discriminated against.

Manning and White[10] studied the attitudes of employers and found that most were cautious about employing currently mentally ill persons, especially those diagnosed as schizophrenics. In its Position Statement on Discrimination, the APA mentions that information about diagnosis or treatment has been used unjustly to deny professional or occupational license and employment and thus reduce opportunities for full participation in society. The narratives reveal that most men in both the groups reported tiredness and inability to work as before. As a result, their family members and spouses ridiculed them more. This could have been because manual labor was common amongst the rural population, which was probably strenuous for those having negative symptoms; whereas lowered self-esteem was frequently reported amongst the urban sample. The urban population avoiding their mental illness histories in job applications significantly more than the rural people could have been due to the nature of jobs held. Significantly more of the rural population were unemployed or did manual labor and looked after their fields and hence formal job applications were not required. The present study measures difficulty in the occupational functioning in people who are already employed. What it does not measure is the difficulty people with illness have in getting a job, as indicated by the fact that 43 of the respondents were unemployed. But the findings are comparable to earlier studies in that the urban population studied does avoid history of mental illness in their application. This could be due to fear of rejection or not being offered a job on disclosing their illness. The APA mentions that “screening” questions in the job interview significantly increase the risk of discrimination; it would be useful to identify these questions and know whether such questions are being asked in India.

Weiss *et al.*,[25] studying psychiatric stigma across cultures, point out that in Bangalore the main concerns the sample had were related to lowering their own chances of entering a good marriage and decreasing the chances of one of their relatives. Raguram *et al.*[24] showed the nature of stigma, as reported by the caregivers, involves getting the patient married, problems in ongoing marriage, and problems for a relative to marry. Similar findings are seen by Thara *et al.*,[21] where many women were separated but not lawfully and did not receive any maintenance from their husbands. Mothers-in-law too played a key role in this situation. Based on the narrative, difficulty in getting married and ongoing problems with their spouse/in-laws were reported in both study groups. As majority of the patients in the sample were married, there were few people for whom getting married was a concern.

## CONCLUSIONS

In educational programs, information regarding the acute phase of the illness would be beneficial (irritability, common types of delusions, hallucinations, and disturbances in biological and socio-occupational functioning). Awareness regarding “negative” symptoms is also needed to dispel myths that the mentally ill are “lazy.” The ill effects of stigmatization should also be stressed upon, especially in rural areas, where ridicule was more pronounced. Educating the rural population can prove challenging as it is necessary to understand their values, attitudes, and their explanatory models of illness and then initiate awareness campaigns. Job-related issues need to be targeted in urban areas. It may not only help deserving individuals to be employed but also helps in boosting their self-esteem. There is a need for studies to evaluate myths related to marriage in both men and women with mental illness.

The study is cross-sectional and does not measure stigma longitudinally. However, the narrative experiences provide rich qualitative data that supports the key findings. The study has identified issues specific to rural and urban areas that need to be addressed to tackle stigma and discrimination. The community-based sample was small in comparison to the clinic-based sample. With an adequately large sample from the community, stigma and existing barriers to community interventions can be assessed. This will be a crucial link in integrating mental health services with primary health care.

## APPENDIX 1

**Socio-demographic information respondent details**

**NAME:** __________________________________________

1. **In which of the following categories does your age fall? (Read list)**
  1. 15-29 years old
  2. 30-45 years old
  3. 46-60 years old
2. **Gender**
  1. Male
  2. Female
3. **State:** ________________________________________
4. **What is the highest level of education you have completed?**
  1. Illiterate
  2. Elementary or up to and including grade 5
  3. High school (Grade 6-10)
  4. Diploma/College
  5. University
5. **What is your current marital status?**
  1. Married
  2. Separated
  3. Divorced
  4. Widowed
  5. Single, i.e., never married
  6. Not applicable
6. **What is your employment status?**
  1. Employed full-time
  2. Employed part-time
  3. Self-employed
  4. Unemployed
  5. Retired
  6. Student
  7. Homemaker
7. **What is your religious affiliation?**
  1. Hindu
  2. Muslim
  3. Christian
  4. Other
8. **What is your total household monthly income?**
  1. Less than Rs.1000/-
  2. Rs.1000-2500
  3. Rs.2500-5000
  4. Rs.5000-10000
  5. >Rs. 10,000
  6. Don't know
9. **Residence**
  1. Rural
  2. Urban
10. **Family size []**
11. **Family type**
  1. Single
  2. Joint
  3. Nuclear
  4. Living together
12. **Clinical diagnosis of the patient (specify):**
  ____________________________________
13. **Duration of illness**
  1. 6 months-1 year
  2. 1-2 years
  3. >2 years
  4. Not available

**Stigma and discrimination faced by patient**

1. Have you/your relative faced stigma and discrimination due to the illness? (Record verbatim)

If there is no response to the above question, enquire about:

- Has your life changed after you have had the illness?
- Are there things that you experience which others without the illness do not?
- Do you have to hide your illness?
- Many people with similar illness experience shame, ridicule, and discrimination; have you also experienced these?
  1. Yes 2. No

If no, skip question no. 2.

2. If yes, at what stage of the illness was the stigma and discrimination most felt?

**(Record verbatim response)**

3. **Common stigma and discrimination experienced by the patient**

1. Neighbors/society treat differently
2. Ridiculing by others
3. Making offensive comments
4. Discrimination by family members
5. Worried that neighbors would avoid
6. Physical abuse (kicking, beating)
7. Need to hide from relatives
8. Worried about talking about illness
9. Ashamed or embarrassed about it
10. Worried that I would be blamed for illness
11. Difficulty in getting marriage proposals
12. Any other
13. Not applicable

**4. Attitudes of relatives towards you (tick off the relevant responses):**

1. Acceptance
2. Help with medical support
3. Rejection
4. Avoidance
5. Loss of respect for the family
6. Any other

**5. What about visiting, and visits of, friends and relatives?**

1. Visit as usual
2. Visit less frequently
3. Do not know/not applicable
4. Don't visit at all
5. Any other

**6. When people ask you what is wrong with you, what do you tell them?**

**(One reply only)**

1. I am mentally ill.
2. General weakness, bodily aches, and pains
3. Headache and discomfort in head
4. Not feeling well
5. Boredom
6. Avoid telling about mental illness
7. Tension/Depression
8. Other response

**7. How do people react if they come to know that you have got mental illness?**

1. No change in reaction
2. Call me mad
3. Talking badly and teasing
4. Discrimination
5. Appreciation over improvement
6. Curiosity
7. Show pity
8. Any other

**8. In your opinion what aspect of illness creates stigma?**

**(One answer only)**

1. Long-lasting nature of illness
2. Not being able to work
3. Lack of correct knowledge
4. Presence of socially unacceptable behavior
5. Attribution of supernatural causation
6. Any other

**9. a) Do you think the stigma you suffer can be removed? If yes, to what extent?**

1. Entirely removed
2. Partially removed
3. Not possible to remove
4. Cannot say
5. Any other

**b) What are the common strategies that you use or can be applied to fight the stigma and discrimination? (Record all responses) (Yes/No/Don't know)**

1. Involvement in advocacy activities
2. Immediate challenge of the stigmatizing remark
3. Concealment or selective disclosure of illness
4. Involvement with other consumers
5. Increasing public awareness to reduce the stigma
6. Empathic understanding by others
7. Giving another diagnosis
8. Any other

**10. What are the consequences you have experienced because of the stigma and discrimination?**

1. Avoid disclosing the mental illness histories in jobs/applications
2. Had been turned down for a job in spite of being qualified
3. Not fully accepted in the family
4. Living alone
5. Pushed into unacceptable social situation
6. Sexual harassment
7. Social exploitation
8. Difficulty in renting house
9. Difficulty in finding suitable match for the sibling
10. Difficulty in getting admission to school/college
11. Had to experience poor sexual relationship
12. Any other

**11. Have you been divorced or separated as a result of stigma towards your illness?**

1. Yes
2. No

**12. Nature of stigma experienced: (Write all relevant responses)**

1. Personal area
2. Occupational area
3. Social area
4. Marital life
5. Family life
6. Any other
7. Not applicable

**13. Which of the following do you feel would be most disabling?**

**(One answer only)**

1. Loss of arms or legs
2. Loss of vision/hearing
3. Being permanently bedridden
4. Mental illness
5. Any other

Any other information you would like to share about this topic?

***Thank you***
